# Mass Spectrometry-Based Disulfide Mapping of Lysyl Oxidase-like 2

**DOI:** 10.3390/ijms23115879

**Published:** 2022-05-24

**Authors:** Alex A. Meier, Eden P. Go, Hee-Jung Moon, Heather Desaire, Minae Mure

**Affiliations:** Department of Chemistry, The University of Kansas, Lawrence, KS 66045, USA; aameier@ku.edu (A.A.M.); edenp@ku.edu (E.P.G.); hjmoon@ku.edu (H.-J.M.)

**Keywords:** lysyl oxidase-like 2, lysine tyrosylquinone, disulfide bonds, mass spectrometry

## Abstract

Lysyl oxidase-like 2 (LOXL2) catalyzes the oxidative deamination of peptidyl lysines and hydroxylysines to promote extracellular matrix remodeling. Aberrant activity of LOXL2 has been associated with organ fibrosis and tumor metastasis. The lysine tyrosylquinone (LTQ) cofactor is derived from Lys653 and Tyr689 in the amine oxidase domain via post-translational modification. Based on the similarity in hydrodynamic radius and radius of gyration, we recently proposed that the overall structures of the mature LOXL2 (containing LTQ) and the precursor LOXL2 (no LTQ) are very similar. In this study, we conducted a mass spectrometry-based disulfide mapping analysis of recombinant LOXL2 in three forms: a full-length LOXL2 (fl-LOXL2) containing a nearly stoichiometric amount of LTQ, Δ1-2SRCR-LOXL2 (SRCR1 and SRCR2 are truncated) in the precursor form, and Δ1-3SRCR-LOXL2 (SRCR1, SRCR2, SRCR3 are truncated) in a mixture of the precursor and the mature forms. We detected a set of five disulfide bonds that is conserved in both the precursor and the mature recombinant LOXL2s. In addition, we detected a set of four alternative disulfide bonds in low abundance that is not associated with the mature LOXL2. These results suggest that the major set of five disulfide bonds is retained post-LTQ formation.

## 1. Introduction

**Figure 1:**
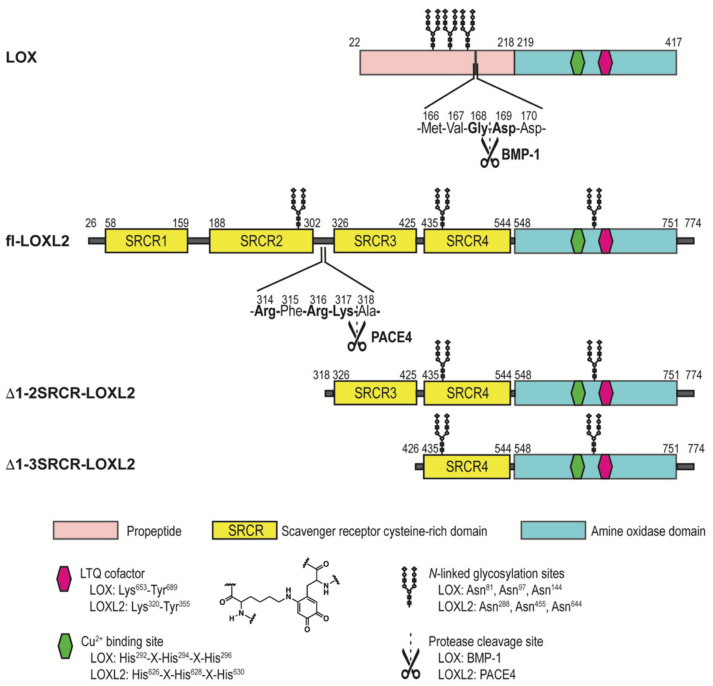
A schematic representation of LOX and forms of LOXL2 used in this study.

**Figure 2:**
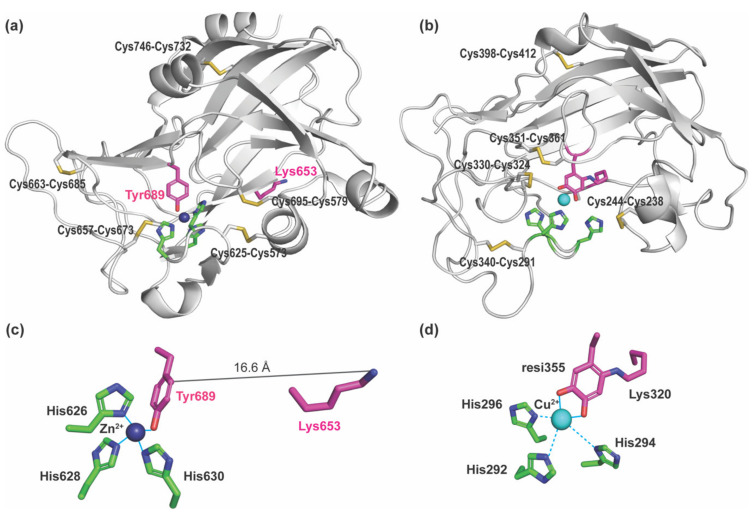
Structures of the Zn(II)-bound precursor form of LOXL2 (PDB: 5ZE3) and the 3D homology modeled Cu(II)-bound mature form of LOX (PDB: ao9b00317_si_002.pdb in [17]). (**a**) The amine oxidase domain of LOXL2. The precursor residues (Lys653 and Tyr689) of the LTQ cofactor are in magenta. A Zn(II) (in navy sphere) occupies the predicted Cu(II) binding site, His626-X-His628-X-His630 motif (in green). There are five disulfide bonds (in yellow) in the amine oxidase domains of LOXL2 and LOX, but four out of five cysteine pairs are different (Table 1). (**b**) The amine oxidase domain of LOX where the LTQ structure (Lys320 + resi355) was modeled in. Cu(II) (in cyan sphere) is near the His292-X-His294-X-His296 motif (in green). (**c**) The ϵ-amino group of Lys653 is located at 16.6. Å distance from C2 of Tyr689 in the precursor LOXL2 structure. Zn(II) is in tetrahedral coordination geometry. (**d**) The o-quinone moiety of the LTQ cofactor is ligated to Cu(II) (2.1 Å, solid lines in cyan) but three His residues are >3 Å away from Cu(II) in the 3D-modeled LOX structure (dashed lines in cyan).

Lysyl oxidase-like 2 (LOXL2) is a Cu(II)- and lysine tyrosylquinone (LTQ)-dependent amine oxidase and belongs to a member of the lysyl oxidase (LOX) family of proteins that catalyze the oxidative deamination of ε-amino groups of peptidyl lysines and hydroxylysines to generate aldehydes (e.g., allysine, hydroxyallysine) [1,2]. The formation of aldehydes initiates crosslinking of the extracellular matrix (ECM) proteins, such as elastin and collagen leading to ECM remodeling. Significant upregulation of LOXL2 has been observed in metastatic/invasive phenotypes of tumor cells and tumors as well as fibrotic disorders [3,4,5,6,7]. Small molecule-based inhibitors of LOXL2 have been under development and some exhibited promising results from in vitro and in vivo studies [8,9,10,11,12,13]. However, due to the lack of structural information on the active site of catalytically-competent LOXL2 containing the LTQ cofactor, the structure-based drug discovery and design targeting LOXL2 has been severely hampered. Recently, Zhang et al reported a 2.4 Å crystal structure (PDB: 5ZE3) of a recombinant human LOXL2, where the first two scavenger receptor cysteine-rich (SRCR) domains at the N-terminus were truncated (Δ1-2SRCR-LOXL2) (Figure 1) [14]. The structure revealed that it is the precursor form (no LTQ), and Zn(II) occupied the predicted Cu(II)-binding site (His626-X-His628-X-His630) (Figure 2a,c). The LTQ cofactor is post-translationally derived from the conserved precursor residues (Lys653 and Tyr689) in the active site of the C-terminal amine oxidase domain (Figure 1). It is proposed that the 1,4-addition of the ϵ-amino group of the Lys653 side chain to dopaquinone, DPQ (the oxidized Tyr689), followed by O_2_ oxidation yield LTQ (Figure S1) [15,16]. However, the mechanism of the LTQ cofactor biogenesis remains elusive. In the precursor structure, Tyr689 was ligated to the Zn(II) taking a part of the tetrahedral Cu(II) coordination geometry. Surprisingly, the ϵ-amino group of Lys653 was located 16.6 Å away from the C2 position of Tyr689 (Figure 2c). Therefore, it was suggested that a substantial conformational rearrangement is required for the biogenesis of the LTQ cofactor [14].

**Figure 3:**
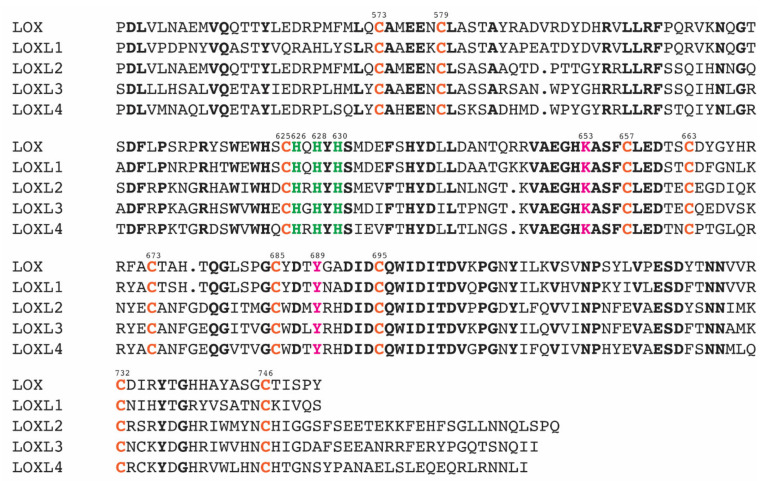
The sequence alignment of the C-terminal catalytic domain of the LOX-family of proteins. The residue numbers are those of LOXL2. Conserved residues are in bold. The His-X-His-X-His motif for Cu(II)-binding is in green. The precursor residues (Lys and Tyr) of the LTQ cofactor are in pink. Conserved Cys residues are in orange. The multiple sequence alignment was conducted by COBALT [19].

The catalytic domains of human LOX (referred to herein as LOX) and LOXL2 share 49% amino acid sequence identity (68% similarity), and within that domain, the three His residues composing the Cu(II) binding site, the LTQ precursor Lys and Tyr residues, and ten Cys residues are completely conserved among the LOX-family of proteins as shown in Figure 3 [1]. Vallet et al. built a 3D model of the amine oxidase domain of LOX by homology modeling based on the Zn(II)-bound precursor LOXL2 structure (Figure 2b) [17] and disulfide bonds determined in the native LOX isolated from bovine aorta [18]. In the cysteine-pairing pattern of the latter, four out of five disulfide bonds were different from those identified in the precursor LOXL2 structure (Table 1). In the modeled LOX amine oxidase domain, the LTQ precursor residues (Lys320 and Tyr355) were close enough to enable the crosslinking of the ϵ-amino side chain of Lys320 to DPQ355. The LTQ cofactor structure was then modeled from the 1.7 Å X-ray structure of the D298K mutant of the copper amine oxidase (CAO) from *Arthrobacter globiformis*, AGAO (PDB: 2YX9) [16], as this is the only available LTQ structure to date. In D298K-AGAO, Asp298 was replaced with Lys by site-directed mutagenesis, and the DPQ intermediate during the topaquinone (TPQ) biogenesis was trapped as LTQ. The resulting modeled LTQ cofactor in LOX is ligated to Cu(II) (2.2 Å) through the *o*-quinone moiety (Figure 2d). Three His residues in the Cu(II) binding site (His292-X-His294-X-His296) are ≥ 3.7 Å away from Cu(II) (His292–Cu(II): 3.7 Å, His294–Cu(II): 4.8 Å, and His296–Cu(II): 5.2 Å) and His296 is proposed to interact with Cu(II) through a water molecule [17]. In the Zn(II)-bound precursor LOXL2, the distances between Zn(II) and three His ligands are 2.0–2.1 Å (Figure 2c), which are very similar to those (2.0–2.2 Å) observed in CAOs (e.g., PDB: 3WA2). 

Subsequently, an alternative homology model was generated for the amine oxidase domain of LOX to retain the cysteine-pairing pattern of disulfide bonds in the precursor LOXL2 structure (Figure 2a) [17]. However, the model did not allow for the LTQ cofactor biogenesis in the LOX catalytic domain. A possibility of disulfide reshuffling in the precursor form was suggested in order to achieve the pronounced conformational rearrangements for the LTQ cofactor biogenesis in LOX and LOXL2 [17].

During the course of this study, we found that the full-length LOXL2 (fl-LOXL2, ~100-kDa) secreted from human embryonic kidney (HEK) cells and breast cancer cells (MDA-MB-231, MCF-7) undergoes proteolytic cleavage of the first two SRCR domains (SRCR1-SRCR2) to yield Δ1-2SRCR-LOXL2 (~60-kDa) by PACE4, a member of the proprotein convertase family of proteins, at ^314^Arg-^315^Phe-^316^Arg-^317^Lys↓^318^Ala (underlined, PACE4 recognition sequence, Figure 1 and Figure S2). This processing affects neither the amine oxidase activity nor the collagen IV crosslinking activity of LOXL2 in vitro [20]. We performed biophysical characterization of mature forms (containing the LTQ cofactor) of fl-LOXL2 and Δ1-2SRCR-LOXL2 in solution and determined their molecular weights, hydrodynamic radii, radii of gyration, and oligomeric states [21]. Both fl-LOXL2 and Δ1-2SRCR-LOXL2 are predominantly monomers, but oligomerization of the fl-LOXL2 to its dimer and tetramer was also detected on a native PAGE gel, in size exclusion chromatography and analytical ultracentrifugation (AUC). On the other hand, no oligomer was detected for Δ1-2SRCR-LOXL2, suggesting that one or both of SRCR1 and SRCR2 at the N-terminus are involved in oligomerization. Importantly, the hydrodynamic radius and the radius of gyration of the mature Δ1-2SRCR-LOXL2 were very similar to the corresponding values that were calculated for the Zn(II)-bound precursor crystal structure, indicating that the overall folds of the precursor and the mature Δ1-2SRCR-LOXL2s are very similar. Based on these results, we proposed that extensive conformational rearrangements are not required for the LTQ cofactor biogenesis.

Mass spectrometry has been widely used for disulfide mapping of proteins. Generally, the sample is prepared under non-reducing conditions to keep disulfide bonds intact [22]. A protein sample is first subjected to alkylation of free Cys residues and deglycosylation. The alkylated and deglycosylated protein sample is then digested by trypsin or other proteases and subjected to liquid chromatography–tandem mass spectrometry (LC–MS/MS). We developed a simple approach for the assignment of disulfide connectivity using extracted ion chromatograms of the electron transfer dissociation (XIC/ETD) method for rapid disulfide bond analysis in proteins [23]. This method can be utilized for detection of disulfide shuffling and analysis of heterogeneity when more than one disulfide bond pattern exists. The method was successfully applied to define disulfide bonds of a recombinant monomer of the HIV envelope protein, gp120 [23], and IgG3 monoclonal antibodies isolated from human serum [24].

Previously, we have also developed a general protease digestion procedure for the optimal protein sequence coverage and post-translational modifications (PTMs) analysis of recombinant proteins [25]. We applied the procedure to Δ1-3SRCR-LOXL2 (Figure 1) that was isolated from a suspension culture of *Drosophila S*2 Expression System (DES™) for the LTQ cofactor identification and *N*-glycan profiling [25,26]. The LTQ cofactor in Δ1-3SRCR-LOXL2 was covalently modified with phenylhydrazine prior to proteolysis. The sequence coverage after trypsin digestion was 71.3% and cysteine-containing peptides were detected at 77.8%. The phenylhydrazine derivatized LTQ-containing crosslinked peptides were isolated and Lys653 and Tyr689 (in fl-LOXL2 numbering) were confirmed as precursors of the LTQ cofactor [26].

In this study, we conducted mass spectrometry-based disulfide mapping using three different forms of recombinant LOXL2s, namely (1) a mature fl-LOXL2 containing nearly the stoichiometric amount of the LTQ cofactor; (2) a precursor Δ1-2SRCR-LOXL2 (no LTQ); (3) a 1:3 mixture of mature and precursor forms of Δ1-3SRCR-LOXL2. Samples (1) and (2) were isolated from a FreeStyle™ 293-F expression system and sample 3) was isolated from DES™. The domain organization of fl-LOXL2 in the AlphaFold 2 [27,28]-predicted structure (https://alphafold.ebi.ac.uk/entry/Q9Y4K0, accessed on 9 February 2022) is shown in Figure S2 together with the color-coordinated primary amino acid sequences.

The LTQ cofactor in the mature LOXL2 was covalently modified by 2-hydrazinopyridine (2HP), an analog of phenylhydrazine, to block the highly reactive *o*-quinone moiety of the LTQ cofactor during proteolysis and mass spectrometry. Moreover, 2HP was used in this study because of its stability in a buffered aqueous solution as opposed to phenylhydrazine, which undergoes oxidation at physiological pH to produce phenyl radical [29,30]. If our hypothesis (e.g., disulfide bonds are conserved) is correct, for the amine oxidase domain of the mature form, we anticipate detecting fragments containing the same set of five disulfide bonds detected in the precursor crystal structure (Table 1). Although disulfide bonds are conserved, we should also detect fragments unique to the precursor form (trypsin cleaves after Lys653) and the mature form (containing LTQ-2HP and the trypsin cleavage site at Lys653 is blocked). If disulfide shuffling occurs during biogenesis, we expect to observe a set of disulfide bonds in the mature form that is completely different from the precursor form. 

## 2. Results

### 2.1. Quantitation of the LTQ Cofactor in fl-LOXL2 and Δ1-3SRCR-LOXL2

**Figure 4:**
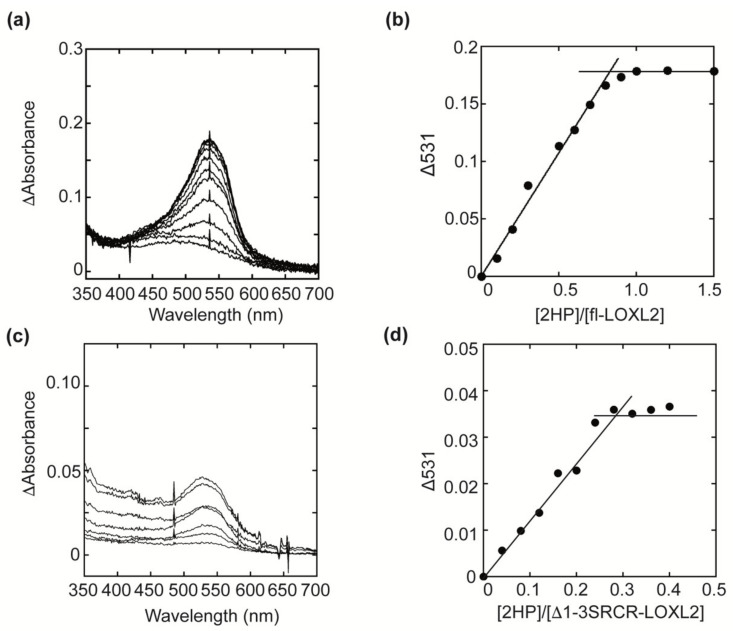
UV-vis spectroscopic titration of the LTQ cofactor by 2HP. (**a**) UV-vis spectral change during the 2HP titration of fl-LOXL2. (**b**) A plot of absorbance change at 531 nm (baseline subtracted) versus the molar ratio of 2HP over fl-LOXL2. (**c**) UV-vis spectral change during the 2HP titration of Δ1-3SRCR-LOXL2. (**d**) A plot of absorbance change at 531 nm (baseline subtracted) versus the molar ratio of 2HP over Δ1-3SRCR-LOXL2.

The UV-vis spectra of fl-LOXL2 and Δ1-3SRCR-LOXL2 before and after titration with 2HP are shown in Figure 4a,c. An increase in absorbance at 531 nm was observed when a semiquantitative amount of 2HP was added sequentially to protein samples at pH 8 (Figure 4b,d). The spectral change at 531 nm saturated around [2HP]/[fl-LOXL2] = 0.95 and [2HP]/[Δ1-3SRCR-LOXL2] = 0.27, respectively, indicating that fl-LOXL2 contains nearly the stoichiometric amount (~95%) of the LTQ cofactor, whereas Δ1-3SRCR-LOXL2 contains 27% of the LTQ cofactor. These results were consistent among several batches of both proteins prepared at different times. Further study is necessary to understand why DES™ did not fully support the LTQ cofactor biogenesis. It is possible that the homologous expression system (FreeStyle™) is more suited to produce a recombinant LOXL2 closely related to the native state than the heterologous expression system (DES™). 

### 2.2. Correlation between the LTQ Content and the Amine Oxidase Activity of fl-LOXL2 and Δ1-3SRCR-LOXL2

**Figure 5:**
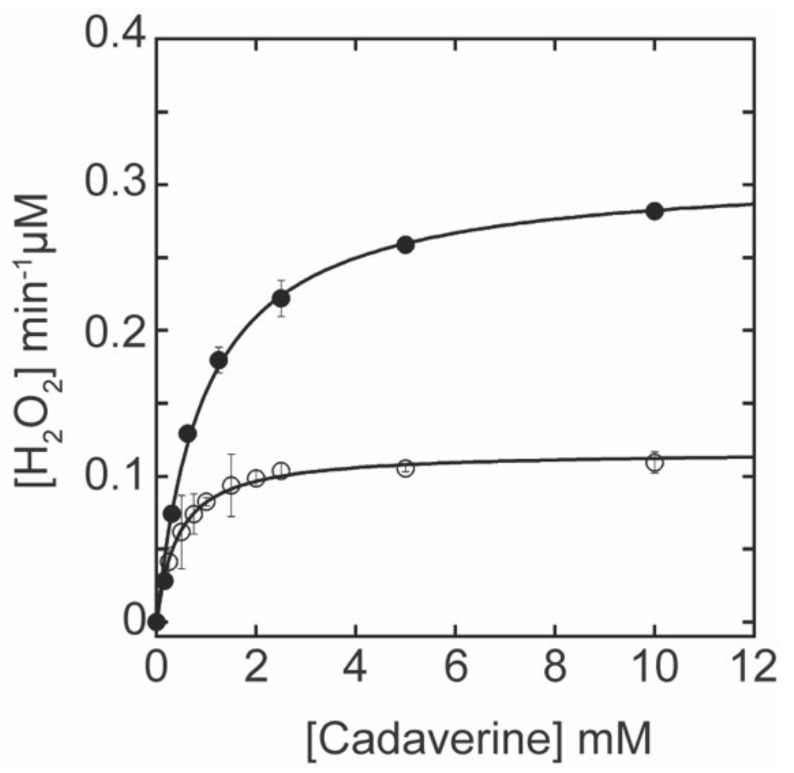
The oxidation of cadaverine by fl-LOXL2 (●) and Δ1-3SRCR-LOXL2 (○) follows the Michaelis–Menten kinetics at pH 8.0. The equation used for non-linear curve fitting to obtain the kinetic parameters is: V = {(*k*_cat_ [S])/((*K*_M_ + [S]) )} × [E_T_], where E_T_: total enzyme concentration, [S]: substrate concentration, *k*_cat_: turnover *K*_M_: Michaelis–Menten constant. The assay was performed in triplicate and data were plotted as the mean of three data points with standard deviation.

In order to determine the correlation between the LTQ content and the amine oxidase activity of Δ1-3SRCR-LOXL2, the standard LOX amine oxidase assay was performed using cadaverine as the assay substrate [20]. Michaelis–Menten kinetic parameters were obtained from the non-linear curve fit analyses and compared to those of fl-LOXL2 (Figure 5). The *k*_cat_ value (catalytic turnover) of Δ1-3SRCR-LOXL2 is 38% of that of fl-LOXL2, reflecting the 27% LTQ in the former. The *K*_M_ value of Δ1-3SRCR-LOXL2 is smaller (≤½) than that of fl-LOXL2. Further study is necessary to understand the origin of the reduction in *K*_M._ Nonetheless, the catalytic efficiency (*k*_cat_/*K*_M_) of Δ1-3SRCR-LOXL2 is comparable to that of fl-LOXL2 (Table 2), supporting our previous observation that the number of SRCR domains does not have a significant impact on the catalytic efficacy of LOXL2 in vitro [20]. Δ1-3SRCR-LOXL2 was chosen for mass spectrometry-based disulfide mapping analysis in this study along with 2HP-inhibited fl-LOXL2 and Δ1-2SRCR-LOXL2 in the precursor form, because it is a mixture of precursor and mature forms and only contains the fourth SRCR domains and C-terminal amine oxidase domain. Since both forms are present within the same batch of the sample, we should be able to detect more than one set of disulfide bonds (cysteine-pairing patterns) if disulfide bond shuffling occurs during the LTQ cofactor biogenesis.

### 2.3. Assignment of Disulfide-Containing Peptides

**Figure 6:**
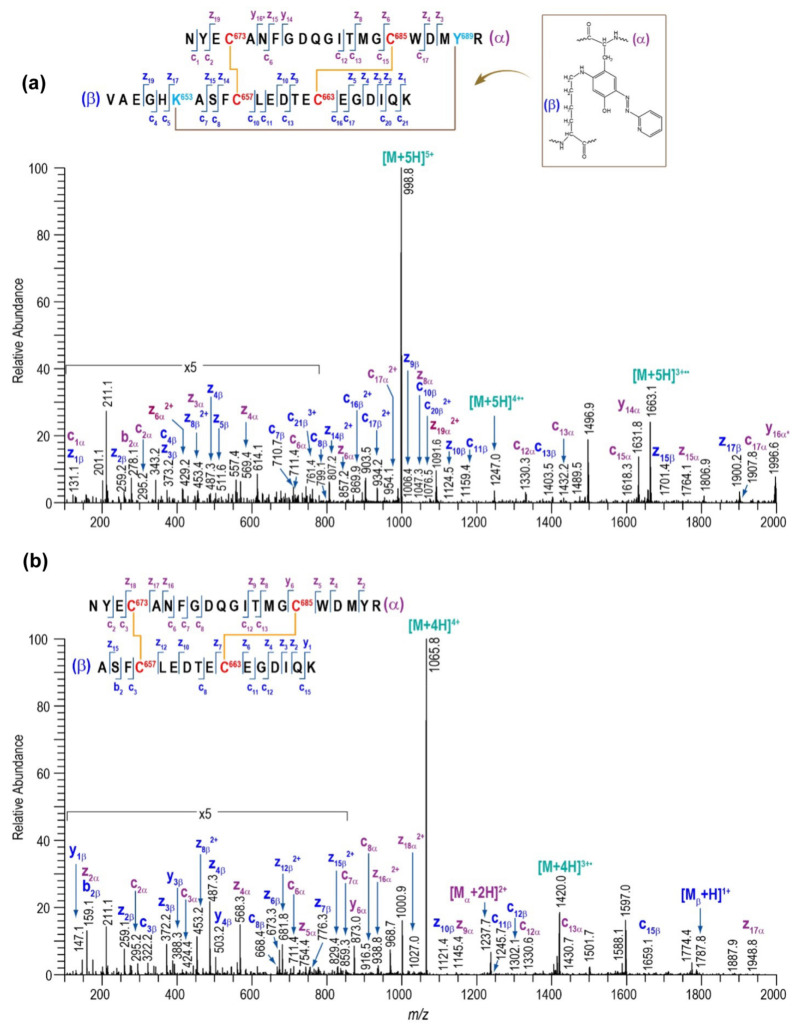
MS/MS data confirming the presence of peptide no. 12. (**a**) ETD data for the peptide containing LTQ cofactor (peptide no. 12b). (**b**) ETD data for the peptide without the LTQ cofactor (peptide no. 12a).

A list of major disulfide-containing peptides identified after digestion of 2HP-inhibited fl-LOXL2 with trypsin and sequential digestion with trypsin and chymotrypsin is summarized in Table 3 and Table S1. We identified all disulfide-containing peptides that were predicted for the instances when the patterns of cysteine-pairing of disulfide bonds in the precursor form were retained in the mature form. We detected two peptides 12a (derived from the precursor form) and 12b (derived from the mature form); the latter contains the 2HP-derivatized LTQ (crosslink at Lys653 and Tyr689) (Figure 6). From our UV-vis spectroscopic titration of fl-LOXL2 with 2HP, the fl-LOXL2 sample contains ≥95% LTQ (Figure 4a,b), so the peptide 12a accounts for ≤5% of the sample; the fact that it was detected clearly demonstrates the method’s appropriateness for identifying low-abundant isoforms. Lists of major disulfide-containing peptides identified after trypsin digestion of the precursor Δ1-2SRCR-LOXL2 and the mixture of Δ1-3SRCR-LOXL2 are summarized in Table 4, Tables S2 and S3. All cysteine-pairing patterns in disulfide bonds are conserved between the precursor and the mature LOXL2 (Table 3 and Table 4), demonstrating that disulfide bond shuffling is less likely to be associated with the LTQ cofactor biogenesis. 

In addition to these major disulfide-containing peptides, additional peptides containing disulfide bonds with distinct cysteine-pairing patterns were also detected for all three forms of LOXL2, where primarily neighboring cysteine residues made pairs (Table 5 and Tables S4–S6). We detected four alternate disulfide bonds in the mixture of Δ1-3SRCR-LOXL2 (Table 6), suggesting that the fifth disulfide bond associated with this sample must be Cys732–Cys746, which is also conserved in between the crystal structure of the precursor LOXL2 and 3D-predicted LOX. Two disulfide bonds (Cys573–Cys579, Cys657–Cys663) with the alternate cystine-pairing patterns match with those disulfides in the predicted LOX structure, but the other two (Cys625–Cys695, Cys673–Cys685) are different from those in the predicted LOX (Cys625–Cys673, Cys685–Cys695) (Table 1). It should be noted that these peptides are in low abundance especially those derived from Δ1-2SRCR-LOXL2 and fl-LOXL2 (Table 6). These low-abundance, alternative disulfide-bonded peptides with neighboring cysteines are commonly seen when other well-folded proteins are subjected to this disulfide-mapping workflow, including those that have been quantified to be >95% natively folded by multiple biophysical methods [31,32]. These peptides, which contain a single tryptic peptide and internal disulfide bond, are likely minor degradation products that happen after tryptic digestion.

**Figure 7:**
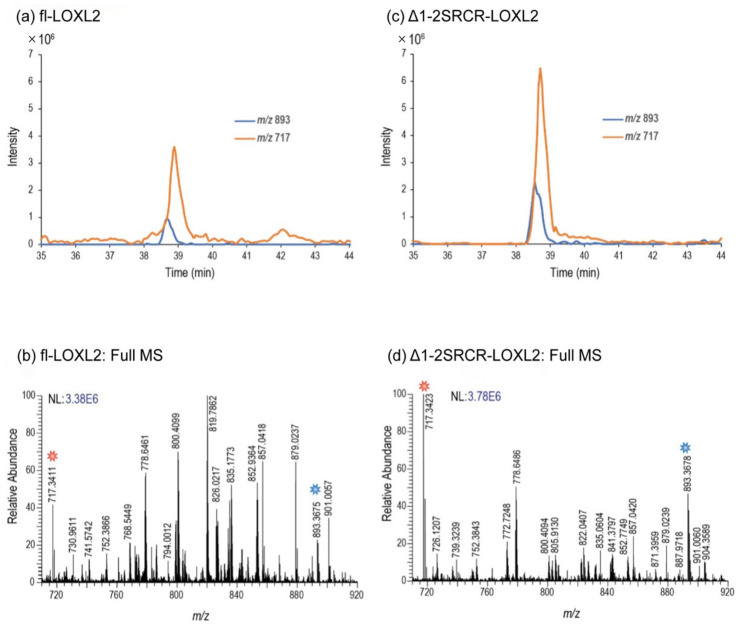
Quantitative comparison peptide no. 18 (sequence is shown in Table 5) in fl-LOXL2 (Table S6) and Δ1-2SRCR-LOXL2 (Table S5). (**a**) The XIC of peptide no. 18 (m/z 893) and a co-eluting peptide (m/z 717), used as an internal standard, for fl-LOXL2. (**b**) The mass spectrum generated from the LC–MS in (a) when both peaks are eluting. (**c**) The XIC of peptide no. 18 and its coeluting internal standard for the Δ1-2SRCR-LOXL2. (**d**) The mass spectrum generated from the LC–MS data in (**c**) when both peptides are present. By comparing the chromatograms in (**a**,**c**), one can clearly observe that the abundance of peptide no. 18 does not appreciably change with respect to the internal standard peptide, m/z 717, when the construct changes from fl-LOXL2 to Δ1-2SRCR-LOXL2.

Using a co-eluting peptide as an internal standard, we verified that no appreciable change in the abundance of the disulfide-linked peptide no. 18 containing Cys657–Cys663 (in Table 6) occurs when comparing the precursor Δ1-2SRCR-LOXL2 and the 2HP-inhibited mature fl-LOXL2, further verifying that this disulfide bonding configuration does not form as part of LTQ biogenesis (Figure 7). These results reveal that this alternate cysteine-pairing pattern is not associated with the LTQ biogenesis. Further study is necessary to determine whether this alternate cysteine-pairing is an artifact of recombinant protein expression, the analysis method, or if it exists in native LOXL2 (e.g., secretes from MDA-MB-231 cells).

## 3. Discussion

By mass spectrometry-based disulfide mapping, we identified a set of five disulfide bonds both in the precursor (no LTQ) and 2HP-inhibited mature (containing LTQ) LOXL2s. This set of disulfide bonds matches with the set that was detected in the Zn^2+^-bound precursor LOXL2 structure (Table 1), suggesting that the cysteine-pairing patterns of disulfide bonds are retained during the LTQ biogenesis. In addition, we also detected four alternate disulfide bonds in low abundance that were not associated with 2HP-inhibited mature LOXL2. Two of the four disulfide bonds are in the 3D-modeled LOX that is based on disulfide bonds identified in the native bovine LOX [18]; however, the two other bonds are different. It should be noted that we detected the set of alternate disulfide bonds containing peptides more in Δ1-3SRCR-LOXL2 (produced in the heterologous expression system) than Δ1-2SRCR-LOXL2 and fl-LOXL2 (produced in the homologous expression system). It is possible that a form of recombinant LOXL2 containing an alternate set of disulfide bonds accounts for the observed insufficiency for the LTQ biogenesis in Δ1-3SRCR-LOXL2 as this set is not associated with the LTQ cofactor.

An important question remains to be answered. How does the precursor LOXL2 overcome the 16.6 Å distance observed in the Zn^2+^-bound crystal structure to support the formation of the LTQ cofactor? The recent 3D-modeled structure of mouse LOXL2 (AlphaFold V2: https://alphafold.ebi.ac.uk/entry/P58022, accessed on 21 January 2022) may provide us with a plausible explanation. The C-terminal amine oxidase domains of mouse and human LOXL2s share 90.7% sequence identity (www.expasy.org/SIM, accessed on 3 March 2022). In the predicted mouse LOXL2 structure, the set of five disulfide bonds matches that detected in the Zn^2+^-bound precursor structure (Figure S3a, Table 1). Lys655 (Lys653 in human LOXL2) is a part of a loop as opposed to a part of the short β8 (pentapeptide) detected in the latter and resides within 4.7 Å of the C2 position of Tyr691 (Tyr689 in human LOXL2) (Figure S3b). Most likely this part of the peptide containing Lys653 of LOXL2 has some conformational flexibility to enable the 1,4-addition of the ε-amino group of Lys653 to DPQ (activated Tyr689) in the solution (Figure S1).

## 4. Materials and Methods

### 4.1. Materials

All reagents and solvents were purchased at the highest purity available. Cadaverine dihydrochloride, 2HP dihydrochloride, horseradish peroxidase (HRP), ethanol, 4-vinylpyridine, ammonium citrate, and citric acid were purchased from Sigma-Aldrich (St. Louis, MO, USA). Boric acid, sodium borate, and N-acetyl-3,7-dihydroxyphenoxazine (Amplex™ Red Reagent) were obtained from Thermo Fisher Scientific (Lenexa, KS, USA). Optima™ LC/MS-grade acetonitrile, water, and formic acid were purchased from Fisher Scientific (Thermo Fisher Scientific). Sequencing grade trypsin, chymotrypsin, and glycerol-free peptidyl-*N*-glycosidase F (PNGase F) were purchased from Promega (Madison, WI, USA) and New England BioLabs (Ipswich, MA, USA), respectively. Deionized water with a resistivity of 18 MΩ∙cm was purified using a Millipore Direct-Q3 Water Purification System (Billerica, MA, USA). 

### 4.2. Preparation of LOXL2 Samples for Mass Spectrometry-Based Disulfide Mapping

The mature fl-LOXL2 and the precursor Δ1-2SRCR-LOXL2s were both produced in FreeStyle™ 293 Expression System (Thermo Fisher Scientific) in the presence and absence of CuSO_4_, respectively. Both proteins were purified as described previously [20], except for the precursor Δ1-2SRCR-LOXL2 for which all buffers and solutions used for protein purification contained 1 mM EDTA, and at the last step, EDTA was removed by dialysis with a 30K MWCO membrane (Millipore, Burlington, MA, USA). Δ1-3SRCR-LOXL2 was produced in DES™ (Thermo Fisher Scientific) and purified as described previously [26]. 

### 4.3. HP Titration of LTQ in Mature LOXL2

The LTQ cofactor in the mature fl-LOXL2 and the mixture of Δ1-3SRCR-LOXL2 was titrated with 2-HP by UV-vis spectrometrically on an Agilent 8453 UV-Vis spectrophotometer. An aliquot of 2HP (0.1 molar equivalent to LOXL2) was added to LOXL2 (350 μg of fl-LOXL2, 180 μg of Δ1-3SRCR-LOXL2) in 50 mM of HEPES buffer pH 8.0 in a stepwise fashion. The reaction was incubated at 25 °C in a capped cuvette until no further spectral change was observed (15 min) after each aliquot addition of 2HP. The UV-vis absorbance was corrected for dilution effects. Data analysis was performed using Kaleidagraph v.4.5.4. (Synergy Software, Reading, PA, USA). 

### 4.4. Amine Oxidase Activity Assay of LOXL2

Amine oxidase assays were performed as previously described [20]. Briefly, the assay was conducted on a Synergy H3 (BioTek, Winooski, VT, USA) in the kinetics mode with the excitation wavelength at 544 nm and the emission wavelength set to 590 nm to monitor the formation of resorufin. A 40 nM solution of LOXL2 was prepared in 50 mM of borate buffer pH 8.0 containing 50 μM of Amplex red and 5 mU/mL of HRP. This assay mixture was pre-incubated at 37 °C for 10 minutes before the addition of 10 μL of cadaverine at varied concentrations. The relative fluorescent unit (RFU) over time (min) was plotted and the slope of the linear range (ΔRFU/min) was converted to (H_2_O_2_)/min via a standard curve.

### 4.5. Protease Digestion LOXL2 Proteins for Disulfide Analysis

Disulfide bond patterns of LOXL2 were determined by mapping the disulfide-linked peptides by mass spectrometry. About 40 μg of LOXL2 samples were alkylated with a 10-fold molar excess of 4-vinylpyridine in the dark for one hour at room temperature to cap free cysteine residues. Alkylated LOXL2 samples were subsequently deglycosylated with 500 U of PNGase F for one week at 37 °C. The fully deglycosylated and alkylated samples were digested with trypsin and a combination of trypsin and chymotrypsin. Protease digestion was performed according to the manufacturer’s suggested protocols: digestion with trypsin was performed overnight with a protein to enzyme ratio of 30:1 at 37 °C; for the combination of both proteases, samples were digested using a protein:enzyme ratio of 30:1 (trypsin) and 20:1(chymotrypsin) and were incubated overnight at 37 °C. The digests were either directly analyzed or stored at −20 °C until further analysis.

### 4.6. Chromatography and Mass Spectrometry

High-resolution LC/MS experiments were performed using an Orbitrap Fusion Tribrid (Thermo Scientific) mass spectrometer equipped with ETD that was coupled to an Acquity UPLC M-Class system (Waters, Milford, MA, USA). Mobile phases consisted of solvent A: 99.9% deionized H_2_O + 0.1% formic acid and solvent B: 99.9 % CH_3_CN + 0.1% formic acid. Three microliters of the sample were injected onto the C18 PepMap™ 300 column (300 μm i.d. × 15 cm, 300 Å, Thermo Fisher Scientific) at a flow rate of 3 μL/min. The following CH_3_CN/H_2_O multistep gradient was used: 3% B for 2 min, followed by a linear increase to 30% B in 50 min, to 55% in 15 min, then to 90% B in 25 min. The column was held at 97% B for 10 min before re-equilibration. The mass spectrometric analysis was performed in the positive ion mode using a data-dependent acquisition with the instrument set to run in 3-sec cycles for the survey and two consecutive MS/MS scans either with CID and EThcD/ETciD or with EThcD and ETciD. The full MS survey scans were acquired in the Orbitrap in the mass range, 350–1800 *m/z* at a resolution of 120,000 at *m/z* 200 with an AGC target of 4 × 10^5^ and a maximum injection time of 50 ms. Following a survey scan, MS/MS scans were performed on the most intense ions with intensities greater than 1000. CID was carried out with a collision energy of 35% while ETD was performed using the calibrated charge-dependent reaction time. The resulting fragments were detected using a rapid scan rate in the ion trap.

### 4.7. Disulfide Analysis of LOXL2 Proteins

Disulfide bond patterns of LOXL2 proteins were determined by mapping the disulfide-linked peptides. Data analysis was performed using the Mascot (v. 2.7) search engine for peptides containing free cysteine residues; disulfide bond patterns were analyzed manually as described previously [33,34]. Briefly, to determine peptides containing free cysteine residues, raw data generated from LC/MS/MS experiments were converted to MGF format using MSConvert. The MGF files were then searched against the UniProt Swiss-Prot database (2021_02 release, 564,638 sequences, 203,519,613) using Mascot v. 2.7.0 (MatrixScience, Boston, MA, USA). The following search parameters were used: enzyme used: either trypsin alone or a combination of trypsin and chymotrypsin, a maximum miscleavage of per peptide, a mass tolerance of 10 ppm for precursor, and ± 0.6 Da for fragment ions; amino acid modifications that were used: fixed: pyridylmethyl(Cys); variable: deamidation (N/Q), Gln-›pyro-Glu(N-term Q), and oxidation (M). An automatic decoy search was applied to determine the false discovery rate (FDR) and when possible, peptides were evaluated at 1% FDR. The Mascot ion score cut-off was 21. Using these parameters, no peptide was identified with free cysteine, which indicates that all cysteine containing peptides are disulfide-bonded. To this end, all disulfide-bonded peptides were analyzed manually. 

## 5. Conclusions

In this study, we defined the cysteine-pairing patterns of LOXL2 both in precursor and 2HP-inhibited mature forms by mass spectrometry-based strategies. The major cysteine-pairing patterns of disulfide bonds in the C-terminal amine oxidase domain of precursor LOXL2 completely match with those detected in the crystal structure of the precursor form. Further, this cysteine-pairing pattern is conserved in the mature LOXL2, strongly suggesting that the major set of disulfide bonds detected in the precursor LOXL2 is retained in the 2-HP inhibited mature LOXL2. In addition, we also detected disulfide bonds with an alternate cysteine-paring pattern in all three forms of LOXL2, where primarily neighboring cysteine residues made pairs. However, these peptides are in low abundance, and their presence is not related to the LTQ cofactor’s biogenesis, because they are present in the same quantity in both fl-LOXL2 and Δ1-2SRCR-LOXL2. Interestingly, among these peptides, two are in the 3D-modeled LOX structure but the other two alternate disulfide bonds detected have different cysteine-pairing patterns. Importantly, we did not detect any LTQ-2HP in these alternate disulfide bonds, further suggesting that this disulfide bonding configuration does not support the LTQ cofactor biogenesis.

## Figures and Tables

**Table 1:** Disulfide bonds in the amine oxidase domains of LOX and Δ1-2SRCR-LOXL2.

LOXL2 ^1^	LOX ^2^	LOX ^3^
Cys573–Cys625	Cys238–Cys244	**Cys573–Cys579**
Cys579–Cys695	Cys291–Cys340	**Cys625–Cys673**
Cys657–Cys673	Cys324–Cys330	**Cys657–Cys663**
Cys663–Cys685	Cys351–Cys361	**Cys685–Cys695**
Cys732–Cys746	Cys398–Cys412	Cys732–Cys746

^1^ Observed in the crystal structure of a Zn(II)-bound precursor Δ1-2SRCR-LOXL2 [16]. ^2^ Predicted in the 3D homology-modeled structure of LOX [19] retaining the cysteine-pairing pattern of the four identified disulfide bonds in the native LOX isolated from bovine aorta [18]. ^3^ Cysteine pairings of disulfide bonds and cysteine residue numbers that are based on LOXL2. In bold: different patterns of cysteine pairings of disulfide bonds observed in the modeled structure [19].

**Table 2:** The Michaelis–Menten kinetics parameters for cadaverine oxidation by fl-LOXL2 and Δ1-3SRCR-LOXL2.

LOXL2	LTQ (%) ^1^	*k*_cat_ (min^−1^)	*K*_M_ (mM)	*k*_cat_/*K*_M_ (min^−1^mM^−1^)
fl-LOXL2	95	7.74 ± 0.19	0.96 ± 0.08	8.08 ± 0.69
Δ1-3SRCR-LOXL2	27	2.93 ± 0.04	0.43 ± 0.03	6.86 ± 0.44

^1^ The LTQ contents were determined by UV-vis spectroscopic titration with 2HP (Figure 4).

**Table 3:** Major disulfide bonds identified for the 2HP-inhibited fl-LOXL2.

Peptide (#)	Domain	Disulfide	Disulfide-Linked Peptides
1	1st SRCR	Cys84–Cys148	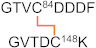
2		Cys97–Cys158	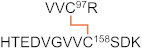
3		Cys128–Cys138	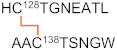
4	2nd SRCR	Cys218–Cys291 + Cys231–Cys301	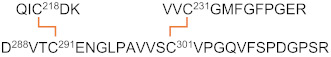
5		Cys265–Cys275	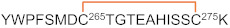
6	3rd SRCR	Cys351–Cys414	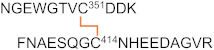
7		Cys364–Cys424	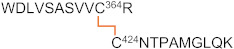
8		Cys395–Cys405	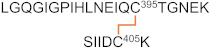
9	4th SRCR	Cys464–Cys530 + Cys477–Cys543	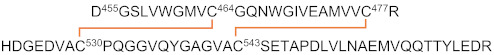
10		Cys511–Cys521	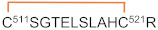
11	Amine oxidase domain	Cys573–Cys625 + Cys579–Cys695	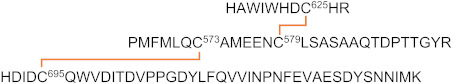
12a 12b		Cys657–Cys673 + Cys663–Cys685	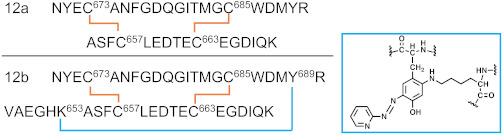
13		Cys732–Cys746	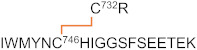

**Table 4:** Major disulfide bonds identified for the precursor Δ1-2SRCR-LOXL2 and the 2HP inhibited mixture of Δ1-3SRCR-LOXL2.

Peptide (#)	Domain	Disulfide	Δ1-2SRCR-LOXL2	Δ1-3SRCR-LOXL2
			Precursor	Mixture
6	SRCR3	Cys351–Cys414	✓	−
7		Cys364–Cys424	✓	−
8		Cys395–Cys405	✓	−
9	SRCR4	Cys464–Cys530 + Cys477–Cys543	✓	✓
10		Cys511–Cys521	✓	✓
11	Amine oxidase domain	Cys573–Cys625 + Cys579–Cys695	✓	✓
12		Cys657–Cys673 + Cys663–Cys685	12a	12a + 12b
13		Cys732–Cys746	✓	✓

The peptide number (#) is from Table 3.

**Table 5:** Additional disulfide bonds detected for the mixture of Δ1-3SRCR-LOXL2.

Peptide (#)	Domain	Disulfide	Disulfide-Linked Peptides
14	4th SRCR	Cys464–Cys477	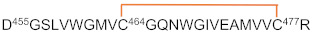
15		Cys530–Cys543	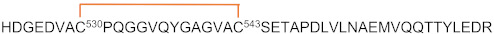
16	Amine oxidase domain	Cys573–Cys579	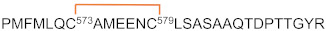
17		Cys625–Cys695	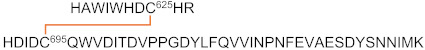
18		Cys657–Cys663	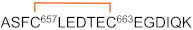
19		Cys673–Cys685	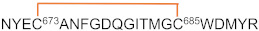

**Table 6:** Additional disulfide bonds identified for the mixture of Δ1-3SRCR-LOXL2, precursor Δ1-2SRCR-LOXL2, and 2HP-inhibited fl-LOXL2. The fragment number (#) is from Table 5. ND: not detected.

Peptide (#)	Domain	Disulfide	Δ1-3SRCR-LOXL2	Δ1-2SRCR-LOXL2	fl-LOXL2
14	SRCR4	Cys464–Cys477	✓	✓	✓
15		Cys530–Cys543	✓	ND	ND
16	Amine oxidase domain	Cys573–Cys579	✓	✓	ND
17		Cys625–Cys695	✓	ND	ND
18		Cys657–Cys663	✓	✓	✓
19		Cys673–Cys685	✓	✓	✓

## Data Availability

The data presented in this study are available upon request from the corresponding authors.

## Supplementary Materials

The following supporting information can be downloaded at: https://www.mdpi.com/article/10.3390/ijms23115879/s1.

## Abbreviations

AGAO	*Arthrobacter globiformis* phenylethylamine oxidase
AUC	analytical ultracentrifugation
BMP-1	bone morphogenetic protein-1
CAO	copper amine oxidase
CH_3_CN	acetonitrile
Cys	cysteine
Da	Dalton
DES™	*Drosophila* expression system
DPQ	dopaquinone
ECM	extracellular matrix
ETD	electron transfer dissociation
FDR	false discovery rate
Gln	glutamine
2HP	2-hydrazinopyridine
His	histidine
HRP	horseradish peroxidase
LC	liquid chromatography
Lys	lysine
LOX	lysyl oxidase
LOXL2	lysyl oxidase-like 2
LTQ	lysine tyrosylquinone
MS	mass spectrometry
MS/MS	tandem mass spectrometry
N-term	N-terminus
PTM	post-translational modification
Pyro-Glu	pyroglutamic acid
RFU	relative fluorescent unit
SRCR	scavenger receptor cysteine rich
TPQ	topaquinone
Tyr	tyrosine
XIC	extracted ion chromatograms
