# Supplementary material for: Mass Spectrometry-Based Disulfide Mapping of Lysyl Oxidase-like 2
Source: Int J Mol Sci. 2022 May 24;23(11):5879. doi: 10.3390/ijms23115879 (PMC9180022; doi:10.3390/ijms23115879)
Supplement: Supplementary file 1 [file ijms-23-05879-s001.zip › ijms-1717358-supplementary.pdf]

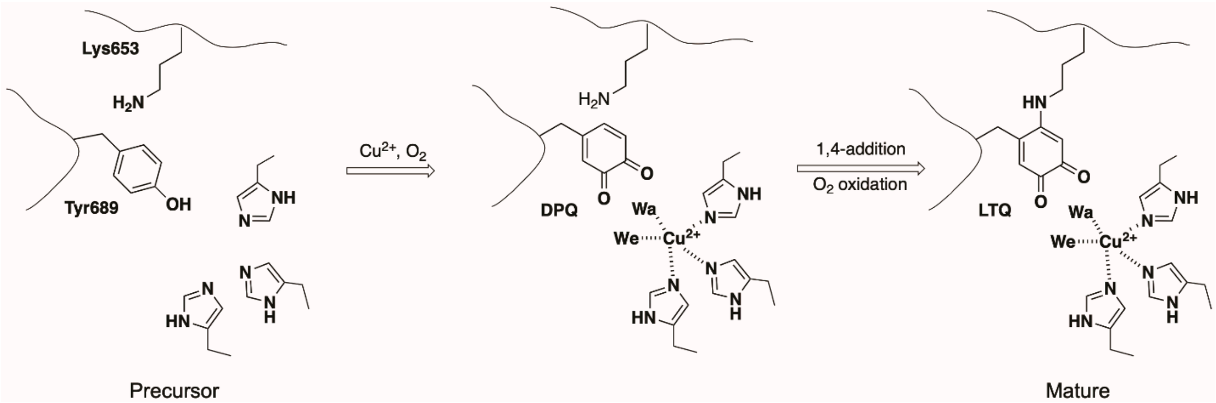

Figure S1. A plausible mechanism for the LTQ cofactor biogenesis from precursor residues (Lys653 and Tyr689). 1,4-Addition of ε-amino group of Lys653 to C2 position of dopaquinone (DPQ) intermediate derived from Tyr689 and subsequent O_2 oxidation yield the LTQ cofactor. The precursor and mature LOXL2s correspond to prior and post LTQ biogenesis, respectively.

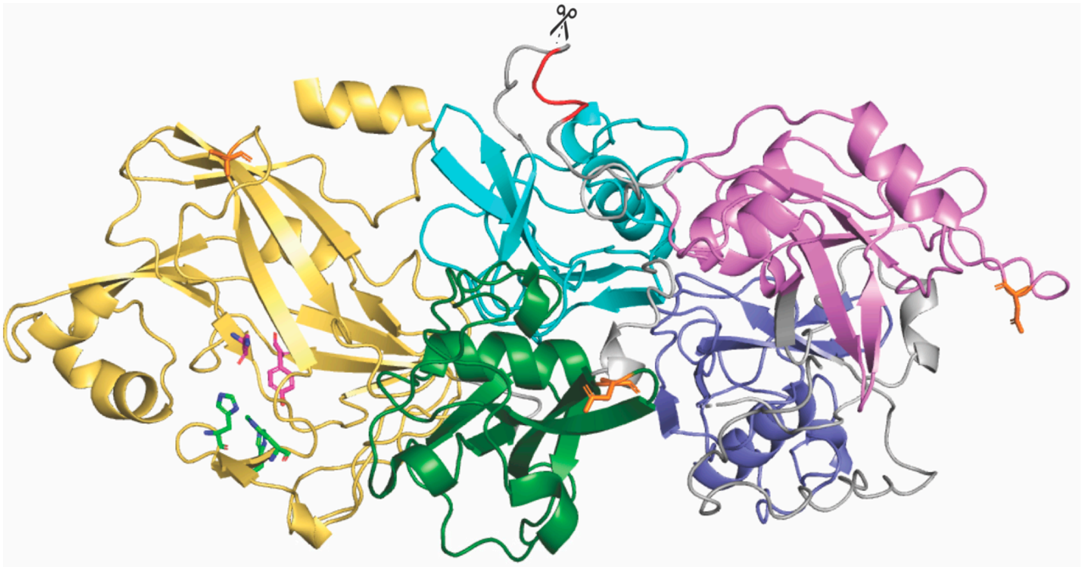

<u>MERPLCSHLCSCLAMLALLSPLSLA</u>QYDSWPHYPEYFQQPAPEYHQPQAPANVAKIQLR
LAGQKRKHSEGRVEVYYDGQWGTVCDDDFSIHAAHVVCRELGYVEAKSWTASSSYGKGE
GPIWLDNLHCTGNEATLAACTSNGWGVTDCKHTEDVGVVCSDKRIPGFKFDNSLINQIE
NLNIQVEDIRIRAILSTYRKRTPVMEGYVEVKEGKTWKQICDKHWTAKNSRVVCGMFGF
PGERTYNTKVYKMFASRRKQRYWPFSMDCTGTEAHISSCKLGPQVSLDPMKNVTCENGL
PAVVSCVPGQVFSPDGPS<u>R</u>F<u>RK</u>AYKPEQPLVRLRGGAYIGEGRVEVLKNGEWGTVCDDK
WDLVSASVVCRELGFGSAKEAVTGSRLGQGIGPIHLNEIQCTGNEKSIIDCKFNAESQG
CNHEEDAGVRCNTPAMGLQKKLRLNGGRNPYEGRVEVLVERNGSLVWGMVCGQNWGIVE
AMVVCRQLGLGFASNAFQETWYWHGDVNSNKVVMSGVKCSGTELSLAHCRHDGEDVACP
QGGVQYGAGVACSETAPDLVLNAEMVQQTTYLEDRPMFMLQCAMEENCLSASAAQTDPT
TGYRRLLRFSSQIHNNGQSDFRPKNGRHAWIWHDCHRHYHSMEVFTHYDLLNLNGTKVA
EGHKASFCLEDTECEGDIQKNYECANFGDQGITMGCWDMYRHDIDCQWVDITDVPPGDY
LFQVVINPNFEVAESDYSNNIMKCRSRYDGHRIWMYNCHIGGSFSEETEKKFEHFSGLL
NNQLSPQ

Figure S2. AlphaFold V2-predicted structure (precursor form) and amino acid sequence of fl-LOXL2 (https://alphafold.ebi.ac.uk/entry/Q9Y4K0). SRCR1: in slate; SRCR2: in light purple; SRCR3: in light blue; SRCR4: in deep green; C-terminal amine oxidase domain: in yellow. *N*-glycosylation sites (Asn288, Asn455, Asn644) are shown in orange. The Cu^{2+}-binding site (His626-X-His628-X-His630) is in green. The precursor residues of the LTQ cofactor (Lys653 and Tyr689) are in magenta. The PACE4 recognition sequence in between SRCR2 and SRCR3 is in red and the cleavage site is indicated by a scissor. Underlined: signal peptide

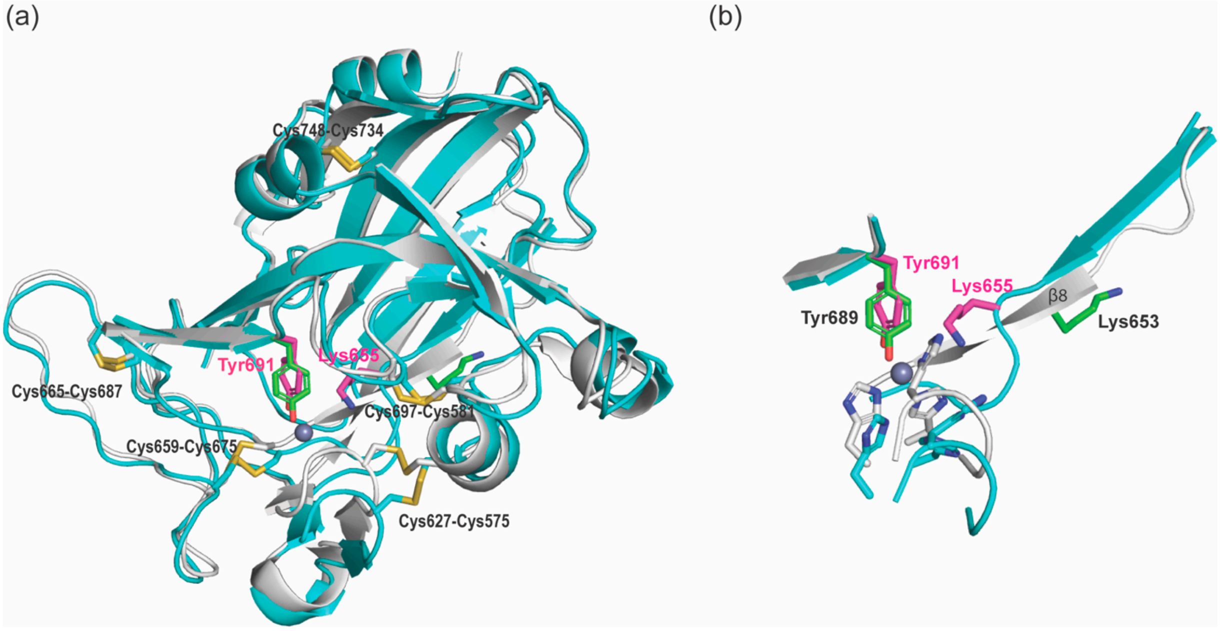

Figure S3. Comparison of active sites of Zn^{2+}-bound precursor human LOXL2 (PDB:5ZE3, in white) and AlphaFold V2 – modeled mouse LOXL2 (https://alphafold.ebi.ac.uk/entry/P58022, in cyan). (a) Superimposition of the C-terminal amine oxidase domains (RMSD: 0.542). Precursor residues for the LTQ cofactor are Lys655 and Tyr 691 (in magenta) for mouse LOXL2 and Lys653 and Tyr 689 (in green) for human LOXL2. The set of five disulfide bonds (Cys575-Cys627, Cys581-Cys697, Cys659-Cys675, Cys665-Cys687, Cys734-Cys748, in yellow) in mouse LOXL2 is completely conserved with that (Table 1) in human LOXL2 (note: residue numbers in mouse LOXL2 are +2 to those in human LOXL2). Some conformational differences are observed for the disulfide bond, Cys575-Cys627, and the loop containing the His-X-His-X-His Cu^{2+}-binding motif. (b) The major conformational/secondary structure differences are detected for Lys655 in mouse LOXL2 (in loop) versus human LOXL2 (in β8). The position of β8 in mouse LOXL2 has shifted towards N-term (e.g. Val650-His654) when compared to His652-Phe656 in human LOXL2.

Table S1. Mass spectrometry data for major disulfide bonds identified for the 2HP inhibited fl-LOXL2.

Peptide (#)	Domain	Disulfide	Charge State	Theoretical *m/z*	Experimental *m/z*	Mass Error (ppm)
1	1st SRCR	Cys84–Cys148	2+<br>3+	745.7924<br>497.5307	745.7931<br>497.5310	1<br>1
2	1st SRCR	Cys97–Cys158	2+<br>3+<br>4+	881.4166<br>587.9468<br>441.2119	881.4169<br>587.9470<br>441.2119	0.4<br>0.4<br>0
3	1st SRCR	Cys128–Cys138	2+<br>3+	876.3593<br>584.5753	876.3603<br>584.5757	1<br>1
4	2nd SRCR	Cys218–Cys291<br>+<br>Cys231–Cys301	3+<br>4+<br>5+<br>6+	1543.7163<br>1158.0390<br>926.6327<br>772.3618	1543.7160<br>1158.0382<br>926.6339<br>772.3624	0.2<br>1<br>1<br>1
5	2nd SRCR	Cys265–Cys275	2+<br>3+<br>4+	1080.9449<br>720.9657<br>540.9761	1080.9446<br>720.9664<br>540.9759	0.2<br>1<br>0.3
6	3rd SRCR	Cys351–Cys414	3+<br>4+<br>5+	1028.4212<br>771.5677<br>617.4556	1028.4212<br>771.5677<br>617.4558	0<br>0<br>0.3
7	3rd SRCR	Cys364–Cys424	2+<br>3+<br>4+	1147.5582<br>765.3745<br>574.2827	1147.5565<br>765.3739<br>574.2820	1<br>1<br>1
8	3rd SRCR	Cys395–Cys405	2+<br>3+<br>4+	1398.7045<br>932.8056<br>699.8559	1398.7030<br>932.8048<br>699.8561	1<br>1<br>0.3
9	4th SRCR	Cys464–Cys530<br>+<br>Cys477–Cys543	5+<br>6+	1449.0581<br>1207.7163	1449.0600<br>1207.7148	1<br>1
10	4th SRCR	Cys511–Cys521	2+<br>3+	637.7845<br>425.5254	637.7845<br>425.5252	0<br>1
11	Amine oxidase domain	Cys573–Cys625<br>+<br>Cys579–Cys695	7+<br>8+	1309.5877<br>1146.0151	1309.5887<br>1146.0156	1<br>0.4
12a<br>12b	Amine oxidase domain	Cys657–Cys673<br>+<br>Cys663–Cys685	12a (precursor)			
			3+<br>4+<br>5+	1419.5630<br>1064.9241<br>852.1407	1419.5626<br>1062.9240<br>852.1400	0.3<br>0<br>1
			12b (mature)			
			4+<br>5+<br>6+	1246.0092<br>997.0088<br>831.0086	1246.0069<br>997.0083<br>831.0083	2<br>1<br>0.3
13	Amine oxidase domain	Cys732–Cys746	3+<br>4+<br>5+	802.6819<br>602.2632<br>482.0120	802.6820<br>602.2633<br>482.0118	0.1<br>0.1<br>1

The peptide number (#) is from Table 3.

Table S2. Mass spectrometry data for major disulfide bonds identified for the precursor Δ1-2SRCR-LOXL2.

Peptide (#)	Domain	Disulfide	Charge State	Theoretical *m/z*	Experimental *m/z*	Mass Error (ppm)
6	3rd SRCR	Cys351−Cys414	2+<br>3+<br>4+	1542.1281<br>1028.4212<br>771.5677	1542.1277<br>1028.4215<br>771.5681	0.3<br>0.3<br>1
7		Cys364−Cys424	2+<br>3+<br>4+	1147.5582<br>765.3745<br>574.2827	1147.5585<br>765.3745<br>574.2831	0.3<br>1<br>1
8		Cys395−Cys405	2+<br>3+<br>4+	1398.7045<br>932.8056<br>699.8559	1398.7040<br>932.8052<br>699.8558	0.4<br>0.3<br>0.2
9	4th SRCR	Cys464−Cys530<br>+<br>Cys477−Cys543	5+<br>6+	1449.0581<br>1207.7163	1449.0586<br>1207.7166	0.3<br>0.3
10		Cys511−Cys521	2+<br>3+	637.7845<br>425.5254	637.7838<br>425.5254	1<br>0
11	Amine oxidase domain	Cys573−Cys625<br>+<br>Cys579−Cys695	6+<br>7+<br>8+<br>9+	1527.6844<br>1309.5877<br>1146.0151<br>1018.7920	1527.6853<br>1309.5882<br>1146.0147<br>1018.7931	1<br>0.4<br>0.4<br>1
12a		Cys657−Cys673<br>+<br>Cys663−Cys685	3+<br>4+<br>5+	1419.5630<br>1064.9241<br>852.1407	1419.5633<br>1064.9248<br>852.1406	1<br>1<br>1
13		Cys732−Cys746	3+<br>4+<br>5+	802.6819<br>602.2632<br>482.0120	802.6827<br>602.2637<br>482.0126	1<br>1<br>1

The peptide number (#) is from Table 3.

Table S3. Mass spectrometry data for major disulfide bonds identified for the mixture of Δ1-3SRCR-LOXL2. The peptide number (#) is from Table 3.

Peptide (#)	Domain	Disulfide	Charge State	Theoretical *m/z*	Experimental *m/z*	Mass Error (ppm)
9	4th SRCR	Cys464−Cys530 + Cys477−Cys543	5+<br>6+	1449.0581<br>1207.7163	1449.0580<br>1207.7161	0.1<br>0.2
10		Cys511−Cys521	2+<br>3+	637.7845<br>425.5254	637.7838<br>425.5254	1<br>0
11	Amine oxidase domain	Cys573−Cys625 + Cys579−Cys695	6+<br>7+<br>8+<br>9+	1527.6844<br>1309.5877<br>1146.0151<br>1018.7920	1527.6855<br>1309.5875<br>1146.0143<br>1018.7931	1<br>1<br>1<br>1
12a<br>12b		Cys657−Cys673 + Cys663−Cys685	12a (precursor)			
			3+<br>4+<br>5+	1419.5630<br>1064.9241<br>852.1407	1419.5623<br>1062.9241<br>852.1402	1<br>0<br>1
			12b (mature)			
			4+<br>5+<br>6+	1246.0092<br>997.0088<br>831.0086	1246.0078<br>997.0094<br>831.0089	1<br>1<br>0.4
13		Cys732−Cys746	3+<br>4+<br>5+	802.6819<br>602.2632<br>482.0120	802.6813<br>602.2631<br>482.0118	1<br>0.2<br>1

Table S4. Additional disulfide bonds detected for the mixture of Δ1-3SRCR-LOXL2.
The peptide number (#) is from Table 5.

Peptide (#)	Domain	Disulfide	Charge State	Theoretical *m/z*	Experimental *m/z*	Mass Error (ppm)
14	4th SRCR	Cys464−Cys477	2+	1304.1025	1304.1008	1
			3+	869.7374	869.7384	1
			4+	652.5549	652.5540	1
15		Cys530−Cys543	3+	1545.6952	1545.6944	1
			4+	1159.5232	1159.5220	1
16	Amine oxidase domain	Cys573−Cys579	2+	1532.6526	1532.6521	0.3
			3+	1022.1042	1022.1043	0.1
			4+	766.8299	766.8294	1
17		Cys625−Cys695	5+	1220.3617	1220.3634	1
			6+	1017.1360	1017.1356	0.4
			7+	871.9747	871.9741	1
18		Cys657−Cys663	2+	893.3689	893.3687	0.2
			3+	595.9150	595.9149	0.2
19		Cys673−Cys685	2+	1236.4792	1236.4782	1
			3+	824.6552	824.6559	1
			4+	618.7432	618.7426	1

Table S5. Additional disulfide bonds detected for the precursor Δ1-2SRCR-LOXL2.
The peptide number (#) is from Table 5.

Peptide (#)	Domain	Disulfide	Charge State	Theoretical *m/z*	Experimental *m/z*	Mass Error (ppm)
14	4th SRCR	Cys464−Cys477[1]	3+	869.7374	869.7377	0.4
16	Amine oxidase domain	Cys573−Cys579	3+	1022.1042	1022.1038	0.4
			4+	766.8299	766.8304	1
18		Cys657−Cys663	2+	893.3689	893.3691	0.1
			3+	595.9150	595.9154	1
19		Cys673−Cys685	2+	1236.4792	1236.4793	0.1
			3+	824.6552	824.6553	0.1

[1] Only one charge state but not the other peptide linked to this peptide was detected due to the low abundance.

Table S6. Additional disulfide bonds detected for the 2HP-inhibited fl-LOXL2.
The peptide number (#) is from Table 5.

Peptide (#)	Domain	Disulfide	Charge State	Theoretical *m/z*	Experimental *m/z*	Mass Error (ppm)
14	4th SRCR	Cys464−Cys477[1]	3+	869.7374	869.7381	1
18	Amine oxidase domain	Cys657−Cys663	2+	893.3689	893.3685	0.4
			3+	595.9150	595.9150	0
19		Cys673−Cys685	2+	1236.4792	1236.4782	1
			3+	824.6552	824.6553	0.1

[1] Only one charge state but not the other peptide linked to this peptide was detected due to the low abundance.
